# Economic Burden of Non-medicinal Poisoning From Healthcare Provider Perspective in 2020: A Prevalence-Based Cost-of-Illness Study in Thailand

**DOI:** 10.34172/ijhpm.8928

**Published:** 2026-01-04

**Authors:** Mu Htay Kywel, Orathai Khiaocharoen, Chatchon Prasertworakul, Tanwa Khattiyod, Sitaporn Youngkong, Arthorn Riewpaiboon

**Affiliations:** ^1^Master of Science Program in Social, Economic and Administrative Pharmacy, Mahidol University, Bangkok, Thailand.; ^2^Thai CaseMix Centre, Division of Healthcare Information Standards Service, Health Systems Research Institute, Bangkok, Thailand.; ^3^Division of Social and Administrative Pharmacy, Department of Pharmacy, Faculty of Pharmacy, Mahidol University, Bangkok, Thailand.

**Keywords:** Economic Burden, Non-medicinal Poisoning, Cost-of-Illness, Thailand

## Abstract

**Background:**

Between 2010 and 2019 in Thailand, hospital admissions due to toxic effects of non-medicinal substances (International Classification of Diseases 10th Revision [ICD-10] codes: T51-T65) ranged from 59.78 to 87.47 per 100 000 population. The objective of this study was to estimate the costs of non-medicinal poisoning from healthcare provider perspective, and identify factors associated with the costs in Thailand for the year 2020.

**Methods:**

This was a prevalence-based cost-of-illness study conducted from healthcare provider perspective, analysing data from five hospitals (four regional and one provincial) across the Central, North, and Northeast regions of Thailand. We included all patients diagnosed with non-medicinal poisoning (ICD-10 codes: T51-T65) during the fiscal year 2020. Direct medical costs were calculated from hospital databases, estimating the cost per outpatient/emergency visit and the cost per hospital admission. Multiple regression analysis was used to determine the factors affecting these costs. All total costs were converted to international dollar (Int$) for 2020.

**Results:**

A total of 3260 patients were included (2472 outpatient visits and 788 admissions). The mean age was 39 years, with 51% being male. The mean cost per outpatient visit was Int$ 47, and the mean cost per admission was Int$ 896. Key factors significantly associated with higher costs included patient type (outpatient vs admission), length of stay (LOS), age, insurance scheme, diagnosis group, and the presence of comorbidities.

**Conclusion:**

This study provided critical, updated data that can inform health policy by emphasizing the economic burden of non-medicinal poisoning. These findings underscore the need for strengthening poisoning prevention and early intervention services and offer essential data for conducting future economic evaluation studies of relevant interventions in Thailand.

## Background

**Key Messages**

**Implications for policy makers**

The findings from this study proved the burden of non-medicinal poisoning in Thailand. The following recommendations on policy-makers are proposed:

- Review and update the National Lists of Essential Medicines continuously with new antidotes added and removed others for adequate evidence of efficacy.
- Advocate the manufacture and distribution of antidotes which are not yet available on the local market, in cooperation with local poison centres, and promote the exportation of these antidotes.
- Develop the production and dissemination of educational materials, including materials targeted at specific high-risk groups, to be properly adapted by all centres for local use.
- Implement mechanisms for mandatory notification of poisoning incidents to public health authorities.
- Encourage to add more interventions in performing further economic evaluation studies in the future.

**Implications for the public**

As the economic burden of non-medicinal poisoning has a significant impact on the healthcare system, public awareness for the initial management, treatment and the information about poisoning centres should be enhanced. Understanding the risks and costs of non-medicinal poisoning encourages safer, more responsible use of household chemicals and agricultural products. On the other hand, education grants and advanced trainings on toxicology for healthcare staffs working in poison treatment units should be offered. In conclusion, funding, public awareness, and education programs can reduce the incidence and economic burden of non-medicinal poisoning while fostering a safety-conscious community.

Currently, non-medicinal poisoning is one of the most under-recognized and under-reported healthcare problems around the world. According to the International Classification of Diseases 10th Revision (ICD-10), the non-medicinal poisoning group (T51-T65) includes the toxic effects of alcohol, organic solvents, halogen derivatives of aliphatic and aromatic hydrocarbons, corrosive substances, soaps, and detergents, metals, other inorganic substances, carbon monoxide, other gases, fumes, and vapours, pesticides, noxious substances eaten as seafood and other food items, contact with venomous animals and plants, aflatoxin and other mycotoxin food contaminants, as well as other specified and unspecified substances.^1^

 Depending on separate socio-cultural and environmental risk factors, non-medicinal poisoning patterns vary in several geographic regions. Every year, there are approximately 193 000 deaths resulting from preventable chemical exposures.^2^ As reported by the World Health Organization (WHO) 2018 data addendum, 1.6 million lives and 45 million disability-adjusted life-years were lost in 2016 due to selected non-medicinal poisonings.^3^ This is higher than the 2016 report, which accounts for an estimated 1.3 million lives and 43 million disability-adjusted life-years lost in 2012^2^. While the WHO estimate includes both acute and chronic health effects from chemical exposures, this study focuses specifically on the immediate healthcare costs of acute non-medicinal poisoning episodes. The increase in emergency visits and hospital admissions due to non-medicinal poisoning has been proved in many literatures.^4-7^

 Thailand is also inevitably affected by this public threat. As the government emphasized on industrial growth, many policies have been adopted and developed during the last few decades.^8^ At the same time, chemical use has been rising gradually because of rapid growth in different sectors of the country. During 2010-2019, rates of admission from the toxic effects of substances (ICD-10 codes: T51-T65), were in the range of 59.78 and 87.47 admissions per 100 000 population for the whole country.^9^

 When it comes to clinical management, WHO supports the International Program on Chemical Safety- INTOX project (IPCS-INTOX project). This program provides all countries with access to and shares information about poison treatment, management, and data storage. WHO also created the iCAPS program (Initiative for Coordinated Antidotes Procurement in the South-East Asia Region) to ensure the attainability of essential antidotes and antivenoms in emergencies or on a regular basis in all Southeast Asia countries.^10^ In Thailand, Ramathibodi Poison Centre has great capacity to provide a comprehensive range of poisoning services with reliable resources, and it was officially appointed as the WHO Collaborating Centre for the Prevention and Control Poisoning for all Southeast Asia regions.^10^ As a part of the Thai health system, the National Health Security Office (NHSO) provides financial support to the centre.

 Due to the huge magnitude of both the clinical and economic problems of non-medicinal poisoning, public health policy on disease management should be well-planned based on evidence. In this case, evidence on economic burden is pivotal to priority setting and exploring economic benefits to society in terms of savings from the health interventions, particularly the poison centre. Although Thailand has adequately prepared clinical services, the economic evidence on non-medicinal poisoning to inform national decision-makers is supposed to be lacking. Therefore, this study aims to estimate the costs of non-medicinal poisoning in Thailand in 2020 from healthcare provider perspective and to identify factors associated with increased costs.We hypothesized that cost drivers would include patient demographics (eg, age), comorbidity status, poisoning type, and hospitalization characteristics (eg, length of stay [LOS] and services received).

## Methods

###  Study Design, Sites and Population

 This non-experimental observational study was designed as a prevalence-based cost of illness study from healthcare provider perspective. This study captures all costs associated with existing cases within the defined study period (year 2020) from healthcare provider perspective; therefore, direct medical costs were included.

 The voluntarily participating hospitals were from a disease-specific cost analysis project.^11^ The hospitals were composed of two regional hospitals from the Central, two regional hospitals from the Northeast and one provincial hospital from the North of Thailand. These hospitals were selected based on the availability of relevant secondary data and their ability to provide comprehensive information for the study objectives. While not randomly sampled, they were chosen to provide geographic and institutional diversity to represent different healthcare facility levels across multiple regions of the country.

 All patients diagnosed with ICD-10 codes (T51-T65) as either primary or secondary disease who received services from the outpatient department (including the emergency room) and inpatient department of the study sites in fiscal year 2020 were eligible. The patients with incomplete medical records such as LOS, service utilization and drug information were excluded. Additionally, patients whose medical condition was unrelated to non-medicinal poisoning were excluded as well. ICD-10 code were mapped to poisoning types as follows: alcohol (T51), organic solvents (T52), halogen derivatives of aliphatic and aromatic hydrocarbons (T53), corrosive substances (T54), soaps and detergents (T55), metals (T56), other inorganic substances (T57), carbon monoxide (T58), other gases, fumes and vapours (T59), pesticides (T60), noxious substances eaten as seafood (T61), other noxious substances eaten as food (T62), contact with venomous animals and plants (T63), aflatoxin and other mycotoxin food contaminants (T64), other and unspecified substances (T65).^1^

###  Sample Size Calculation

 Sample size for cost analysis was calculated to verify the models were not underpowered using the following formula^12^:

*n (at least) = 30 *×* IV *

 where n = sample size and IV = number of independent variables.

 There were 11 independent variables, selected from availability in the study hospital databased, included in this study. The potential predictor variables were age, provincial hospital, inpatient type, female, LOS, Civil Servant Medical Benefit Scheme (CSMBS), out of pocket (OOP), Social Security Scheme (SSS), other insurances, group 1 (non-medicinal poisoning as the primary diagnosis without comorbidities) and group 3 (non-medicinal poisoning was present as a comorbidity alongside other primary conditions). Therefore, the minimum sample size was 330.

*n (at least) = 30 × 11 = 330*

 This approach was selected because formal power calculations require prior cost estimates unavailable for non-medicinal poisoning in Thailand. Our sample of 3260 records exceeds this minimum by 9-fold, ensuring robust regression estimates. The precision of cost estimates is reflected in the reported confidence intervals.

###  Data Collection

 Direct medical costs for the whole study period were considered. Patients’ databases and unit costs of medical services at each hospital were provided by the disease-specific cost analysis project.^11^ For each hospital, information was provided in three files: outpatient services (patient details and services per outpatient visit including emergency visit), inpatient services (patient details and services per hospital admission), and unit costs of medical services. The unit cost data included costs on drugs, medical services, medical supplies, laboratory and investigation, and routine services.

###  Data Editing and Cost Calculations

 All medical records of each patient were combined. A data dictionary was conducted, and groups on hospital types, hospital levels, hospital locations, patient types, gender, and insurance schemes were divided and coded. Cost items were regrouped into five categories, ie, drugs, medical supplies, medical procedures, laboratory and investigation services, and routine services. In the drug group, antidotes were selected and matched with references from the Ramathibodi Poison Centre. The ICD-10 codes of patients were sorted and coded into three different groups: Group 1 consisted of patients with non-medicinal poisoning as the primary diagnosis without comorbidities, group 2 included patients who had existing comorbidities when they came for non-medicinal poisoning treatment, and group 3 included patients where non-medicinal poisoning was present as a comorbidity alongside other primary conditions. However, ICD-10 codes that did not relate to non-medicinal poisoning were left out.

 For each hospital, the data file provided an item of service with a quantity in each row. Each service item’s unit cost was matched and calculated. The costs of each item were summed by group of services. Cost per outpatient visit was defined as the total direct medical cost of an outpatient encounter, including all healthcare services provided during the visit. Based on outpatient visit number or admission number, costs of all items with same number were summed to be total costs of each outpatient visit or admission. Then, the cost per outpatient visit and admission were matched by the patient’s hospital number. The cost per outpatient visit or admission depending on different diagnosis group was calculated. Overhead costs and capital costs were excluded from this analysis, consistent with the direct medical cost approach adopted in this study.

 All costs calculated in Thai Baht (THB) were converted into international dollar (Int$) using purchasing power parity (1 Int$ = 12.31 THB in 2020 value).^13^

###  Statistical Analysis

 All data were entered into Microsoft Excel 2019 and Statistical Package for the Social Sciences (SPSS) version 25 was used for statistical analysis.

 Descriptive statistics was used to obtain characteristics of all variables, mean, standard deviation (SD), median, and 95% confidence intervals for numeric variables and percentage of categorical variables. Bivariate statistics was used to determine the difference in costs between hospital levels and diagnosis groups. A *t* test was used to compare costs between provincial and regional hospitals, while analysis of variance was applied to compare costs across the three different diagnosis groups. Multiple regression analysis^14^ using the stepwise method was used to determine factors affecting cost per outpatient visit or admission. Since cost data were not normally distributed, a natural log-transformation was used to meet the criteria of normal distribution. Potential predicting variables were hospital levels, insurance schemes, diagnosis related groups, age, and gender. Model diagnostics^14^ were applied to ensure the quality of cost functions, to confirm a reliable model by verifying that residuals were independent and homoscedastic, and that there was no significant multicollinearity or influential outliers. The predicted cost (in its natural log-transformed form) was formulated by summing the product of each predictor variable’s value and its corresponding unstandardized coefficient from the final multiple regression model. To calculate average cost of each group of patients, the value of each predictor variable was multiplied by the unstandardized coefficient, and all the results were summed. Estimation of forecasted cost was done through the retransformation of log cost using anti-log (exponential) form and then adjusted by the smearing factor.^15^ The smearing factor corrects for the bias that occurs when converting log-transformed predictions back to the original cost scale, assuming normally distributed residuals on the log scale.

 To retransform the log cost, the following equation was applied.^16^


$$E(\cos t)=\left[e^{\left(x_{0}\;\beta \right)}\right][\frac{1}{n}\sum_{i=1}^{n}e^{s_{i}}]$$

Where $\frac{1}{n}\sum_{i=1}^{n}e^{s_{i}}$ = smearing factor; $e^{s_{i}}$ = anti log (exponential) form of unstandardized residual (EXP_Res).

## Results

###  Characteristics of the Patients

 Among the 3260 patients diagnosed with non-medicinal poisoning (ICD-10 codes: T51-T65) included in the study, 2616 (80.2%) received medical services at regional hospitals, while 644 (19.8%) were treated at the provincial hospital. The mean age of the patients was 39 years (SD 22), with a slight majority being male (51%). Most patients in this study were outpatients, while 24% were admitted as inpatients, reflecting the low hospitalization rate. Inpatients had an average LOS of 3 (SD 5) days.

 Patient public health insurance coverage was predominantly the universal health coverage scheme, which was the largest group (67%), followed by the SSS at 12%, and the CSMBS at 9%. Only 8% of patients paid OOP for their healthcare expenses. Among the patients diagnosed with ICD-10 codes: T51-T65, 28% were categorized into group 1 (poisoning as primary diagnosis without comorbidity), 68% were categorized into group 2 (poisoning with pre-existing comorbidity), and the remaining 4% were group 3 patients (poisoning as comorbidity alongside with other primary conditions). The demographic and clinical characteristics of the patients are summarized in Table 1.

**Table 1:** Characteristics of Patients

**Characteristics**		**Provincial Hospital ** **(n = 644)**	**Regional Hospital ** **(n = 2616)**	**Total ** **(N = 3260)**
Age (y), mean (SD)		38 (21)	39 (22)	39 (22)
Length of stay (day) of inpatients, mean (SD)		3 (5)	3 (6)	3 (5)
Gender, No. (%)	Male	376 (58)	1298 (50)	1674 (51)
	Female	268 (42)	1318 (50)	1586 (49)
Patient type, No. (%)	Outpatient	467 (73)	2005 (77)	2472 (76)
	Inpatient	177 (27)	611 (23)	788 (24)
Insurance type^a^, No. (%)	UC	463 (72)	1723 (66)	2186 (67)
	SSS	64 (10)	331 (13)	395 (12)
	CSMBS	60 (9)	236 (9)	296 (9)
	OOP	43 (7)	206 (8)	249 (8)
	Other insurance types	14 (2)	14 (1)	28 (1)
Diagnosis code, No. (%)	Group 1	52 (8)	845 (32)	897 (28)
	Group 2	581 (90)	1641 (63)	2222 (68)
	Group 3	11 (2)	130 (5)	141 (4)
Types of ICD-10 codes (T51-T65), No. (%)	T-54	29 (4)	177 (7)	206 (6)
	T-60	76 (12)	207 (8)	283 (9)
	T-62	69 (11)	288 (11)	357 (11)
	T-63	447 (69)	1712 (65)	2159 (66)
	Other non-medicinal poisonings	23 (4)	232 (9)	255 (8)
Number of patients who received antidotes^b^, No. (%)		44 (7)	159 (6)	203^b^ (6)
Most frequent used antidotes^c^, No. (%)	Activated charcoal	68 (11)	136 (5)	204 (6)
	Antivenoms	0 (0)	80 (3)	80 (2)
	Diazepam	86 (13)	305 (12)	391 (12)
	N-acetylcysteine	237 (37)	302 (12)	539 (17)
	Other antidotes	6 (1)	96 (4)	102 (3)

Abbreviations: CSMBS, Civil Servant Medical Benefit Scheme; OOP, out of pocket; SD, standard deviation; SSS, Social Security Scheme; T-54, Toxic effect of corrosive substances; T-60, Toxic effect of pesticides; T-62, Toxic effect of noxious substances eaten as food; T-63, Toxic effect of venomous animals and plants; UC, universal coverage. *Note*. Group 1: Non-medicinal poisoning without comorbidity, Group 2: Non-medicinal poisoning with comorbidity, Group 3: Non-medicinal poisoning present as a comorbidity alongside other primary conditions. ^a^ 106 patients failed to show their insurance type. ^b^ 203 patients needed antidotes. ^c^ Some patients needed more than one antidote, while some patients were not prescribed any antidotes at all in a given year.

 The most frequent types of non-medicinal poisoning were the toxic effects of venomous animals and plants (T63), accounting for 66% of all cases. This was followed by noxious substances eaten as food (T62) (11%) and pesticides (T60) (9%). Only 6% of all patients received antidotes during their treatment course. N-acetylcysteine (17% of total patients), diazepam (12%), and activated charcoal (6%) were the most frequently used antidotes.

 The unit cost of high-priced antidotes like antivenoms ranged from Int$63 to Int$101, while common antidotes such as activated charcoal, diazepam, and acetylcysteine cost significantly less, ranging from Int$0.4 to Int$3. The unit cost and data sources of drugs, medical services and antidotes is presented in Table 2.

**Table 2:** Unit Cost and Data Sources of Drugs and Medical Services (Int$ in 2020 Values)

**Item**		**Unit**	**Unit Cost**	**Overhead Cost/Capital Cost Included**	**Data Sources**
Drug	Diazepam	5 mg/mL, 2 mL	1	No	Disease-specific cost analysis project
	Activated charcoal	5 g	1		
	N-acetylcysteine	300 mg/3 mL	2		
	Antivenom, Cobra	10 mL	63		
	Antivenom, Green pit's viper	10 mL	66		
	Antivenom, Russel viper	10 mL	63		
	Hemato polyvalent snake antivenom	10 mL	101		
	Neuro polyvalent snake antivenom	10 mL	99		
Lab & investigation	Ultrasound	1 Test	41	No	Disease-specific cost analysis project
	X-ray	1 Test	70		
Medical procedure	Blood transfusion	0 Service	264	No	Disease-specific cost analysis project
	Nursing care	1 Service	48		
	Colonoscopy	1 Service	179		
	Gastroduodenoscopy	1 Service	99		
	Acute haemodialysis	1 Service	284		
	Chronic haemodialysis	1 Service	162		
	Biopsy of skin and subcutaneous tissue	1 Service	162		
	Urinary catheterization	1 Service	7		
	Eye wash	1 Service	6		
	Oxygen	Per day	37		
Routine services	IPD	1 Bed day	19	No	Disease-specific cost analysis project
	OPD	1 Visit	4		

Abbreviations: IPD, Inpatient department; OPD, Outpatient department.

###  Treatment Cost

 The total mean cost per outpatient visit was Int$47 (SD 60), while the mean cost per admission (inpatient) was significantly higher at Int$896 (SD 1857) (Table 3). Outpatient mean cost for regional hospitals (Int$ 48) incurred slightly higher than provincial hospitals (Int$41). For inpatient mean cost, regional hospitals (Int$924) also had higher costs per admission than provincial hospitals (Int$799).

**Table 3:** Cost Per Outpatient Visit or Admission by Hospital Types (Int$ in 2020 Values)

**Cost **	**Provincial Hospital (n = 644)**			**Regional Hospital (n = 2616)**			**Total (N = 3260)**			***P *** **Value**
	**Mean (SD)**	**Median (IQR)**	**95% CI (Lower-Upper)**	**Mean (SD)**	**Median (IQR)**	**95% CI (Lower-Upper)**	**Mean (SD)**	**Median (IQR)**	**95% CI (Lower-Upper)**	
Outpatient										
Drug cost	3 (5)	2 (0-4)	(3-3)	11 (47)	4 (0-9)	(9-13)	10 (42)	3 (0-8)	(8-11)	<.001
Medical supply cost	0 (1)	0 (0-0)	(0-0)	0 (1)	0 (0-0)	(0-0)	0 (1)	0 (0-0)	(0-0)	.031
Medical procedure cost	23 (17)	35 (0-35)	(21-24)	23 (27)	16 (0-32)	(22-24)	23 (25)	17 (0-35)	(22-24)	<.001
Lab & investigation cost	1 (4)	0 (0-0)	(1-1)	3 (30)	0 (0-0)	(2-5)	3 (27)	0 (0-0)	(2-4)	.700
Routine services cost	14 (10)	21 (0-21)	(13-15)	11 (14)	10 (0-11)	(10-11)	11 (14)	10 (0-21)	(11-12)	<.001
Total cost per outpatient visit	41 (31)	58 (0-62)	(38-44)	48 (65)	41 (5-67)	(45-51)	47 (60)	45 (0-65)	(44-49)	<.001
Inpatient										
Drug cost	412 (872)	53 (27-471)	(283-542)	206 (483)	52 (21-223)	(168-245)	253 (599)	52 (22-248)	(211-295)	.004
Medical supply cost	13 (63)	4 (2-5)	(3-22)	19 (52)	1 (0-10)	(15-23)	17 (55)	2 (0-8)	(14-21)	<.001
Medical procedure cost	224 (598)	100 (51-182)	(135-313)	453 (1130)	211 (115-415)	(362-542)	401 (1039)	184 (100-374)	(328-437)	<.001
Lab & investigation cost	45 (88)	28 (17-43)	(32-58)	85 (131)	48 (27-94)	(74-95)	76 (124)	43 (23-79)	(67-85)	<.001
Routine services cost	105 (183)	64 (32-96)	(77-132)	161 (365)	78 (56-170)	(132-190)	148 (333)	78 (38-157)	(125-171)	<.001
Total cost per admission	799 (1,642)	268 (152-866)	(555-1043)	924 (1915)	474 (241-999)	(770-1075)	896 (1857)	431 (223-972)	(765-1025)	.001

Abbreviations: CI, confidence interval; IQR, interquartile range; SD, standard deviation.

 For both outpatients and inpatients, medical procedures and drugs were the largest cost components. Costs of all types of services between provincial and regional hospitals were significantly different (*P*< .05) except those costs of outpatient services for laboratory and investigations (*P*= .700).

 Regarding the costs by diagnosis groups (Table 4), costs increased significantly with the severity of the patient’s condition as indicated by the diagnosis group (*P*< .05). The lowest mean cost was for group 1 (non-medicinal poisoning as primary diagnosis without comorbidity) at Int$ 151 (SD 408). The mean cost nearly doubled for group 2 (non-medicinal poisoning with comorbidity) at Int$283 (SD 1124), and highest cost was for group 3 (non-medicinal poisoning as a comorbidity alongside a primary condition) at Int$395 (SD 1140). This difference was statistically significant for all cost categories (*P*< .05) except for routine services (*P*= .18).

**Table 4:** Cost Per Outpatient Visit or Inpatient Admission by Different ICD Groups (Int$ in 2020 Values)

**Cost Per Outpatient Visit or Admission**		**Group 1 (n = 897)**	**Group 2 (n = 2222) **	**Group 3 (n = 141) **	**Total (N = 3260)**	***P *** **Value**
Drug cost	Mean (SD)	41 (143)	79 (366)	81 (204)	68 (314)	<.001
	Median (IQR)	7 (2-14)	5 (0-16)	27 (5-53)	6 (0-17)	
	95% CI (lower-upper)	(31-50)	(63-94)	(47-115)	(17-79)	
Medical supply cost	Mean (SD)	2 (10)	5 (32)	9 (32)	4 (28)	.006
	Median (IQR)	0 (0-0)	0 (0-0)	0 (0-0)	0 (0-0)	
	95% CI (lower-upper)	(1-2)	(4-6)	(3-14)	(0-5)	
Medical procedure cost	Mean (SD)	61 (226)	131 (606)	197 (706)	114 (536)	<.001
	Median (IQR)	16 (0-36)	35 (0-94)	31 (20-100)	31 (0-60)	
	95% CI (lower-upper)	(45-74)	(105-156)	(79-314)	(59-132)	
Lab & investigation cost	Mean (SD)	16 (65)	21 (68)	38 (146)	21 (72)	<.001
	Median (IQR)	0 (0-18)	0 (0-11)	0 (0-29)	0 (0-15)	
	95% CI (lower-upper)	(11-20)	(18-24)	(14-62)	(15-23)	
Routine services cost	Mean (SD)	32 (82)	48 (198)	71 (206)	44 (174)	.180
	Median (IQR)	10 (0-57)	11 (0-28)	10 (0-57)	11 (0-29)	
	95% CI (lower-upper)	(26-37)	(39-56)	(36-105)	(29-50)	
Total cost per outpatient visit or admission	Mean (SD)	151 (408)	283 (1,124)	395 (1,140)	252 (984)	<.001
	Median (IQR)	45 (17-135)	61 (3-114)	93 (52-278)	60 (17-128)	
	95% CI (lower-upper)	(123-176)	(237-330)	(205-585)	(111-285)	

Abbreviations: CI, confidence interval; IQR, interquartile range; SD, standard deviation. Group 1: Non-medicinal poisoning without comorbidity, Group 2: Non-medicinal poisoning with comorbidity, Group 3: Non-medicinal poisoning present as a comorbidity alongside other primary conditions.

###  Analysis of Factors Affecting Cost 

 The potential predictor variables in the model are presented in Supplementary file 1 (Table S1).

 For analysis of model assumptions and diagnostics, the leverage value was 0.002, which is below the cut-off of 0.01 and indicates no influential observations among the independent variables. The studentized deleted residual was 1.004, confirming the absence of outliers in the dependent variable, based on the recommended threshold of ±2 (or ±3 to 4 for large samples). The scatter plots of studentized residuals against the predicted values and all independent variables shows no funnel-shaped pattern, supporting the assumption of homoscedasticity. The Durbin-Watson statistic was 1.818, falling within the acceptable range of 1.5-2.5, indicating independence of residuals. Cook’s distance was 0.001, well below the acceptable threshold of <1, suggesting no influential cases affecting model estimates. The condition index was <5.183, which met the criteria <30, demonstrating no evidence of multicollinearity.

 To forecast costs of patients with different characteristics, the fitted values of different patient types were estimated with the smearing factor of 1.36. Based on the cost model, the values of interesting factors were varied while the average values of other factors were applied.

Table 5 presents the statistically significant factors associated with an increase in total cost including inpatient status, LOS, diagnosis type, age, and insurance scheme. The final model demonstrated a good fit with an adjusted R^2^ of 0.655. Conversely, being in group 1 (non-medicinal poisoning without comorbidity) was significantly associated with lower costs.

**Table 5:** The Factors Affecting the Total Cost

**Variables**	**Unstandardized Coefficients**		**t**	***P*** ** Value**	**95% CI**	
	**β**	**SE**			**Lower**	**Upper**
Patient type: IP	1.977	0.041	48.105	<.001	1.897	2.058
LOS (day)	0.114	0.006	19.804	<.001	0.102	0.125
Diagnosis type: Group 1	-0.587	0.036	-16.487	<.001	-0.657	-0.517
Age (y)	0.003	0.001	3.250	.001	0.001	0.004
Insurance type: OOP	0.162	0.062	2.597	<.001	0.004	0.285

Abbreviations: CI, confidence interval; IP, inpatient; LOS, length of stay; OOP, out of pocket; SE, standard error. *Note*. Group 1= Non-medicinal poisoning without comorbidity; Dependent variable = Natural logarithm of total cost per outpatient visit or admission; Adjusted R^2^ = 0.655 (R^2^ = 0.656).

 Based on the fitted model, the predicted mean log-costs for different patients were calculated by summing the products of each predictor and its coefficient:

*LnDMC = 6.412 + 1.977 IP + 0.114 LOS - 0.587 group 1 + 0.003 age + 0.162 OOP*

 This sum represents the predicted log-cost. To convert to the original cost scale, the log-cost was first multiplied by the smearing factor to correct for retransformation bias, and then exponentiated. Then, the predicted cost in THB was converted to Int$. The resulting value represents the predicted mean direct medical cost in the natural scale.

 Predicted cost for those using outpatient services was Int$92, while predicted cost for those using inpatient services was Int$148. The difference between the best and worst-case scenarios based on the model highlights the impact of these drivers: for the best-case scenario, an outpatient in group 1 with insurance other than OOP had a predicted cost of Int$64; for the worst-case scenario, inpatient in group 2 or group 3, admitted for 3 days under OOP insurance coverage, had a predicted cost of Int$174. The full regression model and detailed coefficients are presented in Table 5. Model diagnostics confirmed the reliability of the cost function, meeting assumptions for independence of residuals (Durbin-Watson value of 1.818) and homoscedasticity.

## Discussion

 This study provides new evidence on economic burden of non-medicinal poisoning in Thailand in 2020 by estimating mean cost per outpatient visit (Int$47) and cost per admission (Int$896) from the healthcare provider’s perspective. Beyond measuring the economic impact, these study’s findings contribute important groundwork for assessing implications for healthcare delivery, clinical effectiveness, and policy planning within the national health system.

 The average age of patients (39 years) in our cohort aligns with statistics from the Ramathibodi Poison Centre (2017-2020), which indicated that most poisoning exposures occur between the ages of 20 and 49. Similar age-range patterns have also been reported in other studies.^6^ While some international studies^6,17^ reported a male predominance, most likely due to occupational exposures in industries such as construction and manufacturing, the proportions in our study was nearly balanced.

 A notable finding was the high rate of outpatient utilization, with more than half of cases treated in outpatient departments. This pattern aligns with observations from Singapore^6^ and Chile,^18^ suggesting that the majority of non-medicinal poisoning cases in Thailand are mild to moderate in severity. Correspondingly, the average LOS for admitted patients was 3 days, similar to findings in Sri Lanka,^19^ and shorter than in the study from Turkey.^20^ The shorter LOS observed suggests that most poisoning cases in Thailand are effectively managed with timely supportive care. This efficiency is further supported by the availability and readiness of essential antidotes, which contribute to the rapid stabilization of patients and reduced hospital stay.^21^ Furthermore, socio-cultural norms and well-established family support structures may enable earlier transition of patients to home care, thereby contributing to the comparatively shorter LOS.

 Comparing costs across international studies is inherently complex due to differences in perspectives, study scope, local demographics, and treatment practices. To facilitate a rough comparison, all costs were converted to 2020 Int$ using the exchange rate of each country.^22^ Our estimated mean direct medical cost per patient encounter was Int$252 (Int$47 per outpatient visit and Int$896 per admission). This places our costs within the range observed in developing countries, however, significantly lower than some high-income or specific cohort studies.^23^ For instance, the cost was higher than the societal perspective study from India^24^ (Int$104 per farmer) but lower than the cost from the provider perspective in Sri Lanka^25^ (Int$114) for self-pesticide poisoning, which likely reflects a higher severity of intentional self-harm and intensive care cases. Our findings are also considerably lower than the direct medical cost per pesticide-poisoned patient in South Korea^23^ (Int$1526), which may be attributed to differences in their National Health Insurance reimbursement data structure and the health expenditure patterns of their higher-income citizens.^26^

 In agreement with our hypothesis, the multiple regression analysis showed that inpatient status, age, LOS, and insurance scheme (out of pocket, OOP) were significant factors associated with an increase in total cost. Conversely, being in group 1 (non-medicinal poisoning without comorbidity) was associated with lower costs. Compared to patients in group 1, costs were substantially higher for patients in group 2 (non-medicinal poisoning with comorbidity) or group 3 (poisoning as a comorbidity alongside with other primary conditions). This increase is driven by the necessity for more complicated medical treatments and monitoring required for patients with pre-existing conditions. The increases in cost associated with advanced age reflects the higher likelihood of comorbidity and a corresponding longer recovery time, which leads to increased consumption of medical services over a prolonged hospital stay. Furthermore, patients who paid OOP incurred higher costs than those utilizing public health insurance schemes. This finding suggests that OOP patients might receive drugs or services that fall outside the typical limitations or formularies of their insurance benefit packages, thus driving up the total costs.

 Many studies proved that it was cost-effective to invest in early chemical control and low-cost interventions to prevent non-medicinal poisoning. Economic evaluations conducted in high-income countries focused on occupational chemicals and household products.^27-33^ In contrast, studies from lower- and middle-incomed countries emphasized chemicals used in farms and snakebites.^34-38^ Therefore, this study will provide direct evidence for conducting further economic evaluations of effective non-medicinal poisoning interventions in Thailand.

 Additionally, our findings indicate that only 6% of all patients received antidotes as part of their treatments, with diazepam, activated charcoal, and antivenoms being the most common. This usage is consistent with the overall mild nature of the patient cohort, where supportive care is often sufficient. This result must be considered alongside Thailand’s National Antidotes Program, established in 2010,^39^ to ensure equitable access to essential antidotes and antivenoms. The operational and financial support from the NHSO, coupled with the clinical guidance offered by the Ramathibodi Poison Centre, demonstrates effective centralized procurement and coordinated distribution.^40^ By virtue of these, it has been shown to improve timely access, reduce wastage, and control procurement costs.^39,41^

 The direct medical cost of poisoning cases from this study, including incidence, severity, antidote use, and outcomes can be directly used by the NHSO for cost-effectiveness analyses and to guide future budget decisions for the National Antidotes Program. Furthermore, given the proven cost-effectiveness of investing in early chemical control and prevention – highlighted by numerous economic evaluations globally – our study provides crucial local evidence to support the implementation of effective toxicity intervention policies in Thailand.

 Based on these findings, a comprehensive policy strategy is recommended to further reduce the economic burden of non-medicinal poisoning in Thailand. This includes regularly review and update the National List of Essential Medicines to ensure effective antidotes are available and streamline the approval process for generic affordable antidotes. Collaboration with local poison centres to promote the production, distribution, and accessibility of essential antidotes should be strengthened to address supply chain gaps.^42^ Educational programs and materials should be developed and adapted for high-risk populations, ensuring consistent implementation across regions.^3^ Additionally, a mandatory reporting system for poisoning incidents should be established to improve surveillance, facilitate timely public health responses, and guide resource allocation.^43^ Integrating these public health measures with further economic evaluation studies to provide a practical, evidence-based framework for preventing non-medicinal poisoning and mitigating its economic impact nationwide.

 Still, this study has several limitations that should be considered when interpreting the results. First, this was a prevalence-based cost-of-illness study conducted from the healthcare provider perspective. While it estimates the economic burden on healthcare providers, this approach does not account for long-term disease progression, lifetime treatment costs, out-of-pocket expenditures, or productivity losses,^44^ which results in an underestimation of the total societal economic burden.

 Second, due to data limitations, we could not account for patient clustering within hospitals in the regression model, and no formal uncertainty analysis was conducted. These omissions may affect the precision of the regression estimates and predicted costs.

 Third, while the study included data from five hospitals across three regions of Thailand, this sample may not capture the full variability of treatment practices and costs across all hospitals nationwide, limiting the generalizability of the findings. Furthermore, the dataset also included records up to 2020, and future studies incorporating more recent data will be valuable for capturing post-2020 trends.

## Conclusions

 Non-medicinal poisoning imposes a significant and preventable economic burden on the Thai healthcare system. This study estimated the mean direct medical cost per patient to be Int$252, with inpatient admission costs (Int$896) being notably cost driven. Crucially, key factors associated with increased costs include patient type (inpatient status), age, LOS, and comorbidity status. To mitigate this burden, the government should sustain financial support for the poisoning services while also implementing targeted interventions guided by these cost drivers to improve public literacy, minimize severity, and ensure the economic sustainability of poisoning services nationwide.

## Acknowledgements

 The study team acknowledges the Thai CaseMix Center and Health Systems Research Institute for their data contributions.

## Disclosure of artificial intelligence (AI) use

 Not applicable.

## Ethical issues

 The study was approved by the Institutional Review Board of Faculty of Dentistry/ Faculty of Pharmacy, Mahidol University (COE.No.MU-DT/PY-IRB 2022/016.1003).

## Conflicts of interest

 Authors declare that they have no conflicts of interest.

## Data availability statement

 The raw data supporting the conclusions of this article cannot be made publicly available due to patient confidentiality and institutional data protection policies. However, aggregated data are available from the corresponding author upon reasonable request and with appropriate ethical clearance.

## Supplementary files


Supplementary file 1 contains Table S1.
