# Supplementary material for: Economic Burden of Non-medicinal Poisoning From Healthcare Provider Perspective in 2020: A Prevalence-Based Cost-of-Illness Study in Thailand
Source: Int J Health Policy Manag. 2026 Jan 4;15:8928. doi: 10.34172/ijhpm.8928 (PMC12980026; doi:10.34172/ijhpm.8928)
Supplement: Supplementary file 1 — contains Table S1. [file ijhpm-15-8928-s001.pdf]

Article title: Economic Burden of Non-medicinal Poisoning From Healthcare Provider Perspective in 2020: A Prevalence-Based Cost-of-Illness Study in Thailand

Journal name: International Journal of Health Policy and Management (IJHPM)

Authors' information: Mu Htay Kywel[1], Orathai Khiaocharoen[2], Chatchon Prasertworakul[2], Tanwa Khattiyod[2], Sitaporn Youngkong[3]*, Arthorn Riewpaiboon[3]*

[1]Master of Science Program in Social, Economic and Administrative Pharmacy, Mahidol University, Bangkok, Thailand.

[2]Thai CaseMix Centre, Division of Healthcare Information Standards Service, Health Systems Research Institute, Bangkok, Thailand.

[3]Division of Social and Administrative Pharmacy, Department of Pharmacy, Mahidol University, Bangkok, Thailand.

***Correspondence to:** Sitaporn Youngkong, Email: sitaporn.you@mahidol.ac.th & Arthorn Riewpaiboon, Email: arthorn.rie@mahidol.ac.th

Citation: Kywel MH, Khiaocharoen O, Prasertworakul C, Khattiyod T, Youngkong S, Riewpaiboon A. Economic burden of non-medicinal poisoning from healthcare provider perspective in 2020: A prevalence-based cost-of-illness study in Thailand. Int J Health Policy Manag. 2025;14:8928. doi:10.34172/ijhpm.8928

Supplementary file 1

Table S1. List of potential predictor variables included in multiple regression analysis

Variables	Codes and Values
**Dependent Variable**	
LnDMC	Natural Ln form of direct medical cost
**Independent Variables**	
Age (Year)	Continuous (mean= 39)
Length of stay (Day)	Continuous (mean= 3)
Hospital Type	1= Provincial (20%), 0= Regional (80%)
Patient Type	1= Inpatient (24%), 0= Outpatient (76%)
Gender	1= Female (49%), 0= Male (51%)
Dummy variables for Insurance Type; Universal Health Coverage (67%) as reference	
CSMBS	1= Civil Servant Medical Benefit Scheme (9%), 0= Other
OOP	1= Out of Pocket (8%), 0= Other

SSS	1= Social Security Scheme (12%), 0= Other
Others	1= Other Insurances [a] (1%), 0= Other
Dummy variables for Diagnosis Type; Group 2: Nonmedicinal poisoning with comorbidity (68%) as reference	
Group 1	1= Nonmedicinal poisoning without comorbidity (28%), 0= Other
Group 3	1= Nonmedicinal poisoning as comorbidity (4%), 0= Other

Abbreviations: CSMBS, Civil Servant Medical Benefit Scheme; OOP, Out of Pocket; SSS, Social Security Scheme
